# Genome editing for primary immunodeficiencies: A therapeutic perspective on Wiskott-Aldrich syndrome

**DOI:** 10.3389/fimmu.2022.966084

**Published:** 2022-08-18

**Authors:** Asma Naseem, Zohar Steinberg, Alessia Cavazza

**Affiliations:** Infection, Immunity and Inflammation Research and Teaching Department, Great Ormond Street Institute of Child Health, University College London, London, United Kingdom

**Keywords:** rare diseases, primary immunodeficiency, gene therapy, genome editing, CRISPR/Cas9, Wiskott-Aldrich syndrome

## Abstract

Primary immunodeficiency diseases (PIDs) are a group of rare inherited disorders affecting the immune system that can be conventionally treated with allogeneic hematopoietic stem cell transplantation and with experimental autologous gene therapy. With both approaches still facing important challenges, gene editing has recently emerged as a potential valuable alternative for the treatment of genetic disorders and within a relatively short period from its initial development, has already entered some landmark clinical trials aimed at tackling several life-threatening diseases. In this review, we discuss the progress made towards the development of gene editing-based therapeutic strategies for PIDs with a special focus on Wiskott - Aldrich syndrome and outline their main challenges as well as future directions with respect to already established treatments.

## Introduction

Rare diseases, also known as orphan diseases, represent a very heterogeneous group of disorders that affect a limited number of individuals in the general population. To date, approximately 7000 different types of rare diseases are known worldwide, which altogether account for significant numbers of patients and substantial healthcare costs (1, 2). Most rare diseases are genetic disorders that cause significant disability, substantially affect life expectancy, and impair physical and mental abilities. Taken together, the spectrum poses a widespread and pervasive challenge to the affected individuals and their families (3).

Primary immunodeficiency diseases (PIDs) are rare diseases comprising a group of clinically heterogeneous genetic disorders that undermine the balanced regulation of immunity-related pathways (4, 5). Affected individuals have an increased susceptibility to infectious diseases and non-infectious complications, including allergies, malignancies, and autoimmune manifestations. Thanks to recent advancements in genome sequencing technologies and more widespread accessibility to genetic testing, nearly 440 PIDs have been identified so far (6, 7). These widely varying disorders are mostly monogenic, representing an appealing benchmark to test genetic correction strategies for therapeutic purposes. In this review, we aim to underline the recent advances in the field of gene editing and to explore the potential and applicability, as well as anticipated challenges, of targeted genome editing in the context of a PID, Wiskott - Aldrich syndrome.

## Wiskott-Aldrich syndrome

**Figure 1:**
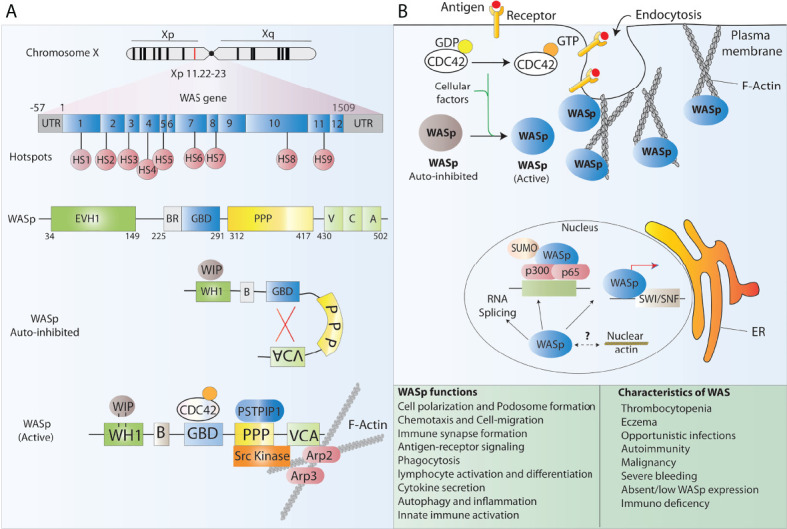
WASp structure and function. **(A)** Schematic representation of the WAS gene and its major functional protein domains. The WAS gene is located on the X chromosome and comprises 12 exons. Disease-causing mutations in WAS patients are scattered around the whole gene, however, 9 mutational hotspots (HS) have been detected in patients. WASp is made of 5 different domains and presents in the cell cytoplasm in an auto-inhibited conformation at resting state and leads to actin polymerization after receptor stimulation. **(B)** The many functions of WASp in lymphoid and myeloid immune cells relate to its role in regulating the polymerization of new branched actin filaments. Besides, WASp plays an important role as a scaffold protein in the regulation of some nuclear functions such as chromatin remodeling.

Wiskott-Aldrich syndrome (WAS) is a rare, X-linked, complex PID caused by mutations in the *WAS* gene which encodes the WAS protein (WASp). The overall incidence of WAS is estimated to be 4 per million live male births, with no ethnic or geographical predominance, making up approximately 3% of all primary immunodeficiencies. The disease was first noticed by the German paediatrician Dr Alfred Wiskott in 1937 when he observed boys dying early in life for three family generations while their sisters being unaffected. Later, Robert Aldrich in the year 1954 confirmed Wiskott’s observations when he tracked and found that 16 out of 40 males in a family died with similar symptoms without affecting females, thus confirming the X-linked mode of inheritance of the disease (8). Several mutations in the WAS gene have been reported that either result in residual or complete absence of WASp in immune cells (**Figure 1A**), leading to a broad heterogeneity of clinical manifestations. Indeed, WAS presents with a very heterogeneous clinical spectrum, ranging from a ‘classical WAS’ form, with severe persistent thrombocytopenia, eczema, increased frequency of opportunistic infections and development of autoimmunity early after birth, to mild asymptomatic thrombocytopenia (X-linked thrombocytopenia [XLT]), or congenital neutropenia (X-lined neutropenia [XLN]). The majority of affected individuals suffer inflammatory complications that impact significantly on quality of life, display an increased incidence of autoimmunity and are at risk of developing lymphoproliferative disorders and lymphoid malignancies (**Figure 1B**). Without a definitive treatment, these patients are not normally expected to survive beyond their second decade (9).

### WASp structure and regulation

WASp is expressed in nearly all hematopoietic cells and belongs to a big family of proteins involved in actin cytoskeleton reorganization and signal transduction. Indeed, WASp is a major effector of actin polymerization and is involved in the reorganization of the actin cytoskeleton together with actin related protein 2/3 complex (ARP2/3) in nonerythroid hematopoietic cells (10) (**Figure 1**). Consequently, WASp deficiency in blood cells affects the actin cytoskeleton integrity and associated cellular processes including cell polarization, podosome formation, cell migration, signalling, autophagy and inflammation among others and results in functional defects in various immune cells, thus impairing innate and acquired immunity (10–14). Several studies have shown that lack of WASp perturbs B cell homeostasis leading to a defective homing and abnormal B cell receptor (BCR) functioning. It has also been reported that a tolerance breach in WASp deficient B-cells due to innate immune activation makes B cells prone to autoantibody development, and that neutrophils further feeds this autoreactivity contributing to the autoimmunity seen in WAS patients (12, 15–17). T cell mediated adaptive immune functions are also compromised in the absence of WASp. WASp deficient T cells present with reduced cytokine production, defective signal transduction and impaired immunological synapse (IS) formation, a structure tightly orchestrated by the actin cytoskeleton, in response to T cell receptor (TCR) stimulation (10, 18, 19) WASp deficiency also perturbs the T cell subset balance that results in lineage skewing from T helper1 (T_H_1) towards T_H_2 dominance (20)). Additionally, WASp deficiency has a profound effect on a variety of functions in innate immune cells such as monocytes, macrophages and dendritic cells (DCs). Macrophages derived from WAS patients fails to assemble podosomes, exhibits phagocytosis defect owing to impaired phagocytic cup formation as well as have abolished chemotaxis, consistent with the pivotal role that WASp plays in actin organization of all aforementioned processes (10, 20, 21). DCs are also affected by WASp deficiency as it has been shown that WASP deficient DCs are unable to form stable IS due to an inefficient priming with T cells (22) Alongside defective immunity, a major hallmark of WAS is microthrombocytopenia with low mean platelet volume, which results in frequent haemorrhages and severe bleeding episodes that lead to death in 4–10% of patients (23) The reasons underlying thrombocytopenia in WAS patients are not fully understood, however, it is speculated that it may be caused either by a reduced platelet production from bone marrow precursors or by an increased clearance of WAS-deficient platelets from the circulation (10)

Structurally, WASp is composed of five major domains which from the amino-terminal to carboxyl terminal are known as a WASp homology 1 (WH1) domain (also known as EVH1, for ENA/VASP homology), a basic (B) domain, a Cdc42 GTPase-binding domain (GBD), a proline-rich domain (PP), and a verprolin homology (V), cofilin homology (C), acidic region (A) (VCA domain) (10) (**Figure 1A**). These functional domains are able to bind and interact with multiple ligands simultaneously and contributes to the dynamic incorporation of actin monomers into filamentous actin (F-actin) to perform crucial cellular functions. The N-terminal WH1 domain of WASp binds to the WASp inhibitory protein (WIP) which is essential to maintain the stability of WASp. Most of the mutations causing WAS are located in this region and it has been shown that disrupting WASp-WIP interaction leads to proteolytic degradation of WASp resulting in WAS *in vivo* (24, 25). Following WH1 region is the B domain which is rich in basic residues and stabilizes autoinhibitory interactions. The GBD contains a Cdc42-Rac-interactive-binding (CRIB) motif, which mediates binding of numerous effectors of the Cdc42 and Rac GTPases. It binds the VCA intramolecularly and plays a key role in controlling activity toward the Arp2/3 complex. The poly-proline domain acts a docking site for multiple SH3 domain containing proteins such as Src and Tec family tyrosine kinases (26).

At resting state, WASp exists in an autoinhibited conformation in which the GBD is bound to the C-region of the VCA domain masking the binding sites for ARP2/3 complex and actin monomers, and hence maintaining the autoinhibition. Upon sensing a stimulus, Cdc42, which is the main and best-characterized activator of WASp, binds to the GBD and the second messenger phosphatidylinositol (4,5)-biphosphate (PIP2) binds to the basic region thereby destabilizing autoinhibitory interactions with the VCA and facilitating the binding of the Arp2/3 complex to the VCA domain leading to WASp activation (9, 26). Through a myriad of interactions, these domains integrate diverse signals to precisely control actin assembly in cells. Few studies have also reported the role of WASp in nuclear functions ranging from regulating nuclear shape to acting as a scaffold protein in chromatin remodelling and gene regulation (27). Control of functional WASp is maintained by phosphorylation of a tyrosine residue (Y291) adjacent to the GBD which marks the protein for ubiquitylation and subsequent degradation (28).

## Established therapeutic approaches for WAS

Adequate supportive treatment with immunoglobulin and antibiotic prophylaxis together with splenectomy enables good survival and quality of life in the short term in patients with WAS. Persistence of infection, bleeding, and autoimmunity and the risk of development of lymphoma, however, suggest the need of a definitive treatment. Given that WAS is a genetic inherited disorder that affects all mature nonerythroid hematopoietic cells, treating this disease requires the restoration of a fully functional hematopoietic system; this could be achieved by: a) infusing normal hematopoietic stem and progenitor cells (HSPCs) derived from healthy donors through hematopoietic stem cells transplantation (HSCT); b) correcting the patient’s own HSPCs. Targeting hematopoietic stem and progenitor cells (HSPCs), which are endowed with self-renewal capacity and differentiation potential, is of paramount importance and could provide to the patients a lifelong benefit with a one-time treatment. The ideal therapy would also need to be applicable to the vast majority of patients affected by WAS, avoiding the need to develop expensive patient-tailored clinical products while providing a “universal” approach that could benefit all WAS patients.

### Hematopoietic stem cell transplantation

As with most PIDs, the standard of care treatment for WAS is HSCT with a busulfan and fludarabine myeloablative conditioning regimen, in combination with palliative care tailored to meet the functioning needs as well as to provide the best possible quality of life for patients (29, 30). The main determinants taken into consideration for a successful HSCT are the donor stem cell source, age of patient at HSCT and pre-HSCT severity of the disease as patients >5years of age, those with a second transplant and those with mismatched allogeneic transplants remain at higher risk, even years after transplant. Although HSCT provides nearly 90% survival rates (31–33), a number of post-transplant complications appear to be higher in WAS, including Graft versus Host Disease (GvHD), toxicity due to the conditioning regimen and autoimmunity, with the latter remaining a significant challenge (34, 35). Occurrence of post-HSCT autoimmunity has been shown in a significant number of patients (8-55%) and its causes are still elusive; this phenomenon could be explained as a result of persistent residual host autoantibodies, or autoantibody-producing plasma cells or lymphocytes which survived ablation by conditioning (29, 36). Thrombocytopenia remains the most difficult WAS defect to cure, with myeloid engraftment rate being the most important predictor of platelet count recovery post HSCT (29, 32).

### Gene therapy

Despite the excellent improvements, there are still many limitations to the implementation of HSCT including: a) short- and long-term complications such as incomplete immune reconstitution, development of autoimmunity, GvHD, deaths and/or long-term dysfunction from the conditioning regimen; b) the need for intense conditioning to achieve successful myeloid and lymphoid engraftment, c) limited availability of HLA-identical family donors (<20% of patients) and racial disparities in accessing matched unrelated donors (37). In addition to “classical WAS’, patients suffering from milder forms of WAS characterized only by thrombocytopenia, such as XLT, represent a much wider cohort of patients that are excluded from HSCT because of a perceived unfavourable risk/benefit ratio; while these patients present with milder phenotypes at diagnosis, they can transition to severe conditions upon development of chronic or life-threatening clinical problems. Thus, safer and alternative treatment options, especially for those children that lack a suitable HSCT donor or that present with a milder form of the disease, are needed.

**Figure 2:**
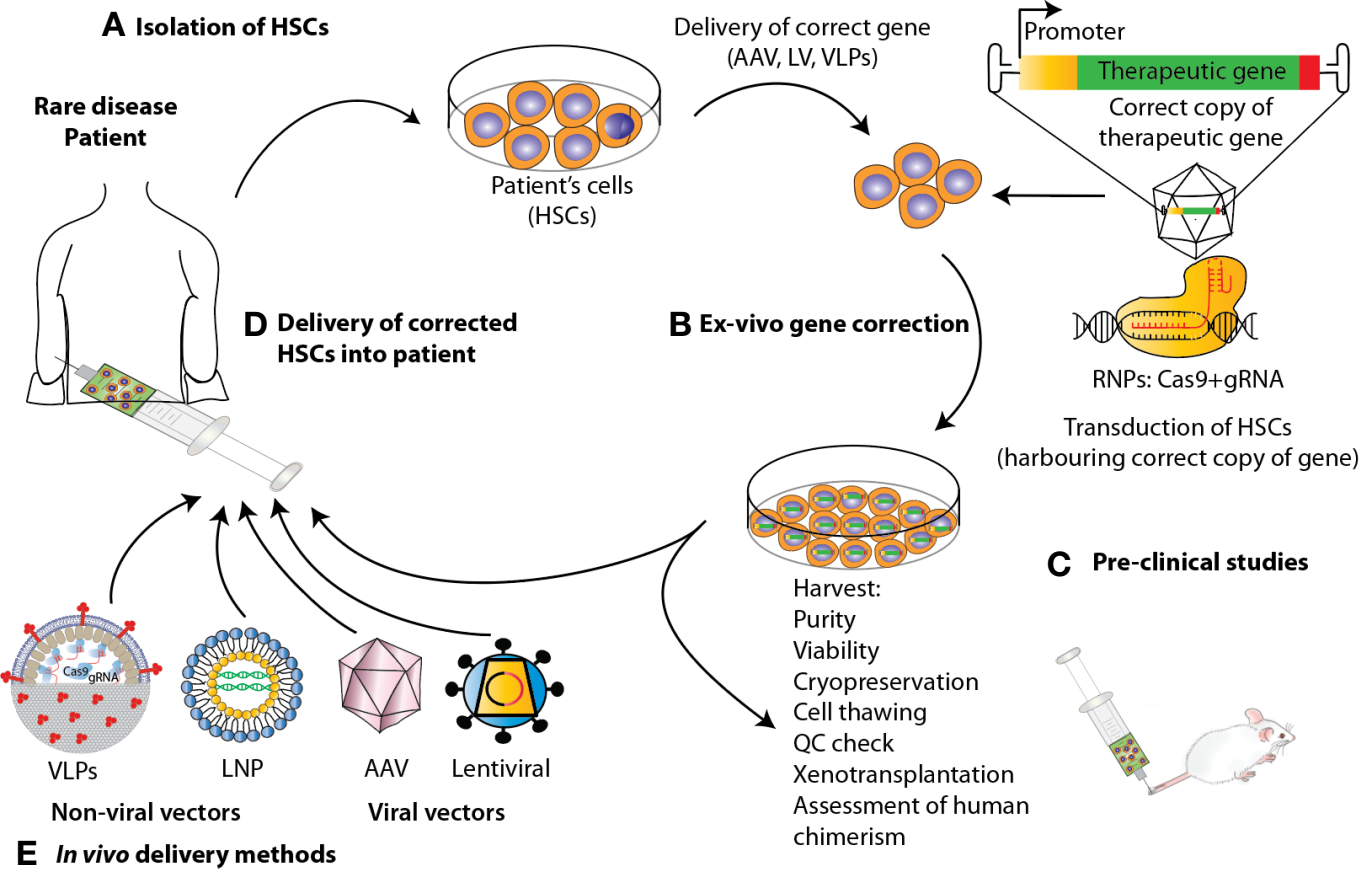
State-of-the-art *ex vivo* and *in vivo* gene therapy and gene editing strategies applied to HSPCs to correct blood genetic disorders *via* delivery of a corrective gene. Schematic representation of the various steps involved in the development of a therapeutic product for the treatment of blood diseases *via* gene therapy. For the *ex-vivo* approach, **(A)** HSPCs are isolated from mobilized peripheral blood (mPB) apheresis. **(B)** Using CRISPR/Cas9-based gene editing or LV carrying the gene of interest, these cells are edited/transduced *ex vivo* and **(C)** after performing a thorough safety and efficacy quality control (QC) on the product **(D)** cells are infused back to the patient after pharmacokinetically adjusted myeloablation. **(E)** When performing gene therapy *in vivo*, the correct copy of the gene is directly infused into the patient *via* the use of viral vectors or non-viral methods such as Virus-like Particles (VLPs) and lipid nanoparticles (LNPs).

Over the past two decades, autologous HSPC gene therapy (GT) has been successfully applied to the treatment of monogenic disorders of the blood. GT utilizing HSPCs is most frequently performed *ex-vivo*, following a process in which bone marrow- or peripheral blood-derived HSPCs are isolated from the patient, genetically-corrected and then reinfused back to the patient where they engraft, undergo self- renewal and establish a population of modified cells that pass the transgene to daughter blood cells on differentiation (38) (**Figure 2**). Genetic correction followed by transplantation of autologous HSCs eliminates the risk of alloreactivity, it is available to those patients that lack a suitable donor for HSCT and facilitates the use of reduced-intensity conditioning, resulting in fewer acute and long-term toxicities. There are several tools that have been developed to mediate gene transfer to target cells. Conventional GT approaches aiming at inserting a correct copy of a gene into HSPCs have fundamentally relied on the use of two main groups of viral vectors, gammaretroviral and lentiviral vectors, with the latter showing the best performance in terms of transduction of quiescent HSCs, efficacy and safety in several clinical trials for primary immunodeficiency and metabolic diseases, aplastic anemia and hemoglobinopathies [reviewed in (38)].

The first GT attempt for WAS dates back to over a decade ago, when 10 paediatric patients were enrolled in a clinical trial in Hannover, Germany. Mobilized autologous CD34+ HSPCs were transduced with a gammaretroviral vector expressing *WAS* under a viral promoter and reinfused back to patients following conditioning. The treatment resulted in excellent engraftment rates and restoration of functionality in all hematopoietic lineages including platelets, with increased platelet counts and termination of bleeding episodes in 9 out of 10 patients (39). Similar to other PID patients treated with gammaretroviral gene therapy, 7 out of these 10 WAS patients developed acute leukemia as a result of the expansion of T cell clones bearing the vector integration sites near the oncogenes *LMO2*, *MDS1* and *MN1* (40). The risk of insertional mutagenesis and oncogene transactivation associated with this generation of GT vectors prompted the development of safer, self-inactivating lentiviral vectors (SIN-LV), hence subsequent clinical trials for the treatment of WAS transitioned to the use of SIN-LV expressing *WAS* under the control of its endogenous promoter, offering a potentially safer and more physiological expression of WASp. Data coming from these studies have confirmed the efficacy of gene therapy for WAS, with no major safety concerns highlighted so far (41–44). An updated report following on the GT trial by Hacein-Abina et al., has shown that in a long-term follow up of WAS GT, gene-corrected cells have engrafted stably with no treatment-associated complications. Importantly, the treatment resolved the severe infections and eczema, while autoimmune disorders and bleeding episodes were significantly reduced but not completely treated (45); this was in line with the partial correction of the platelet compartment, with increased but overall low platelet counts reflective of the lower WASp gene marking in the myeloid versus the lymphoid compartment found in the majority of patients.

Thus, despite the excellent results, the development of alternative and definitive therapeutic solutions is needed for patients with severe WAS and for whom thrombocytopenia is the main complication.

## The rise of nucleases for targeted genome editing

**Figure 3:**
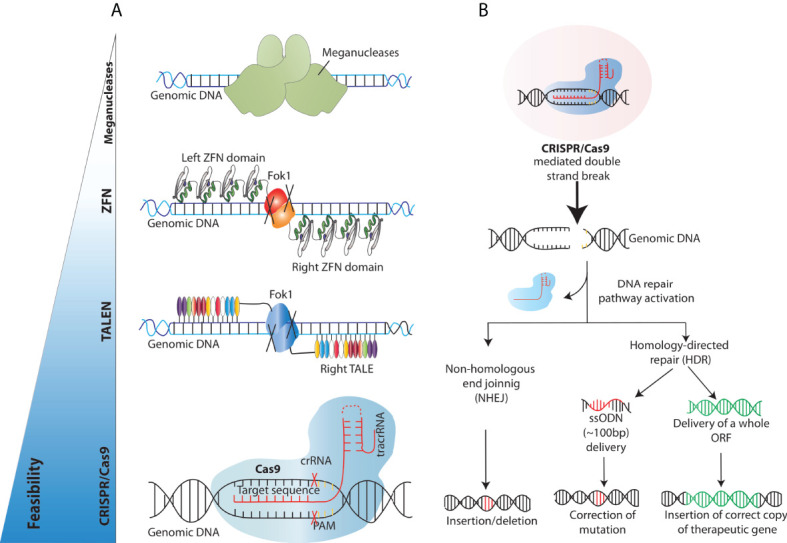
Overview of the existing engineered nucleases platforms and main DNA double-strand breaks repair mechanisms exploited for gene correction. **(A)** Schematics of the different programmable nucleases that have been developed so far **(B)** Engineered nucleases (CRISPR/Cas9 in this case) create DNA double-strand breaks at the genomic site of choice. DNA double strand break repair proceeds either through the non-homologous end joining (NHEJ) or Homology Directed Repair (HDR) pathway. In the NHEJ pathway, random insertions and deletions (indels) are introduced at the cut side and ligated resulting in error-prone repair. In the HDR pathway, the homologous chromosomal DNA exogenously provided serves as a template for the damaged DNA during repair, resulting in error-free correction of a disease-causing mutation if a short ssODN is provided and/or high-fidelity insertion of new genomic sequences, when, for example, a full-length open reading frame (ORF) is provided.

The past decade has witnessed a tremendous progress in the identification and discovery of novel gene editing (GE) tools and precise targeting by means of GE has recently emerged as an alternative technology to overcome the limitations of conventional therapeutic strategies. Genome editing can be carried out by different platforms where programmable DNA nucleases introduce permanent genetic modifications at an established target genomic sequence thanks to the site-specific recognition of DNA sequences. As such, genome engineering tools rely on the combination of two components, a nuclease capable of editing the DNA and a DNA binding region that dictates the specificity of the platform. The initial conceptualization of genome engineering is built on the creation of double-strand breaks (DSBs) at target genomic loci to harness cellular DNA repair mechanisms responsible of inducing the desired DNA edit. These include the error-prone non-homologous end joining (NHEJ) DNA repair pathway and the homology-directed repair (HDR) pathway. The first is a fast and error-prone pathway that ligates the broken DNA ends together without an homologous template and results in random base pair insertions/deletions (indels) and consequently in gene disruption at the target site (46). In contrast, the HDR pathway resolves the DSB with a high-fidelity mechanism that requires the presence of a DNA donor sequence homologous to the region surrounding the DSB (**Figure 3**). Both pathways can be exploited for therapeutic purposes. With NHEJ, indels can be generated to abolish the expression of a protein or the function of a regulatory region and thus neutralize pathological dominantly active genetic element. The most exemplificative application of this approach is the use of gene editing to disrupt the erythroid enhancer for the *BCL11A* transcription factor, a repressor of foetal haemoglobin expression, which yielded promising results in two clinical trials to treat Sickle Cell Disease and β-thalassemia (NCT03655678 and NCT03745287) (47). In contrast, HDR can be utilized to correct a mutation or to insert a specific DNA sequence (single-stranded or double-stranded) at the target site for precise gene editing, using an exogenous donor template harbouring the desired construct. As such, it represents a viable approach to treat those diseases for which correcting or adding a genetic element may bring a therapeutic benefit, like for example WAS.

Over the years, researchers across the globe have focused on developing different tools to achieve targeted DSBs. The techniques used to edit or change the genome have evolved from the earlier attempts of using meganucleases to molecular techniques like transcription activator-like effector nucleases (TALENs) and zinc-finger nucleases (ZFNs) (**Figure 3**). Meganucleases are endonucleases that recognize, and excise long stretches of DNA (~14-40bp). This inherent potential has been manipulated in labs to increase the genome-editing efficiency by modifying the recognition sites to create nicks, a prerequisite for DNA sequence change. These meganucleases are less toxic, as they are naturally occurring and provide precise site cleavage; however, they had a few significant drawbacks. For instance, the probability of finding a meganuclease that targets the desired locus is relatively low. Moreover, and critically, there are high chances of random creation of insertions/deletions (indels) at the break sites (48). These challenges were encountered by discovering and utilising eukaryotic ZFNs, which are composed of zinc ion-regulated small protein motifs coupled to the sequence-independent endonuclease domain of the bacterial restriction enzyme FokI. These ZFNs can bind to DNA in a sequence-specific manner and result in DNA strand breaks at the target sites to increase targeted homologous recombination (49). Although ZFNs proved effective as genome editing tools for some experiments, they were not widely adopted due to the strenuous designing and validation for a specific DNA locus of interest, paving the way for further developments in the field. Soon after, the void was primed by similar but more straightforward and easier to validate transcription activator-like effector nucleases (TALEN) (50, 51). TALENs consisted of tandem arrays of 33–35 amino acids long repeats (~10-30 repeats), with each repeat capable of binding and recognising extended DNA sequences. TALENs are structurally similar to ZFNs as one domain of the TAL repeats is fused to a FokI endonuclease domain, creating a TALEN. Upon binding the two TALENs to flanking DNA sequences, the FokI domains dimerise and introduce a DSB at the target site. Unlike ZFN, TALENs can specifically recognise one base instead of three bases (52–55). Despite their efficiency, these endonucleases presents with some drawbacks such as designing complexity, synthesis, validation, and associated costs, which have limited their widespread adoption for routine use. These obstacles were successfully dealt with the advent of CRISPR/Cas9 technology as it provided better efficiency, feasibility, and multi-role clinical application, thus revolutionising the era of genome editing.

### The advent of CRISPR/Cas

The CRISPR/Cas system was first described by Ishino et al. in 1987 as a series of short, direct repeats interspaced with short non-repeating DNA sequences called spacers in the genome of *Escherichia coli* (56). After a series of discoveries in the following two decades, together with the finding that CRISPR elements are adjacent to multiple well-conserved genes called CRISPR-associated (Cas) genes (57), researchers concluded that CRISPR could serve as a bacterial immune system whose exact mechanism of action was however unknown (58, 59). Later, serial critical findings by various groups further paved the way for CRISPR to become a gene-editing tool. Importantly, Charpentier and colleagues demonstrated that Cas9 enzymes can be reprogrammed to target the desired DNA sequence in bacteria and that the CRISPR/Cas9 can also be guided by a single guide RNA (sgRNA) formed by the fusion of CRISPR RNA (crRNA) and transactivating crRNA or tracrRNA (60–62). Practically, this gRNA sequence can be tailored to optimize hybridization with a particular target site and thereby guide the Cas9–gRNA complex to the site of the desired break (**Figure 3**). The principal difference between CRISPR/Cas9 and the nucleases used in the past is that it is based on the principle of Watson-Crick base pairing, which guides the Cas9/gRNA complex to the target site, making the experimental design relatively simple, highly tailorable and cost-effective (61, 63).

### CRISPR/Cas DSB-free tools: Base and prime editing

**Figure 4:**
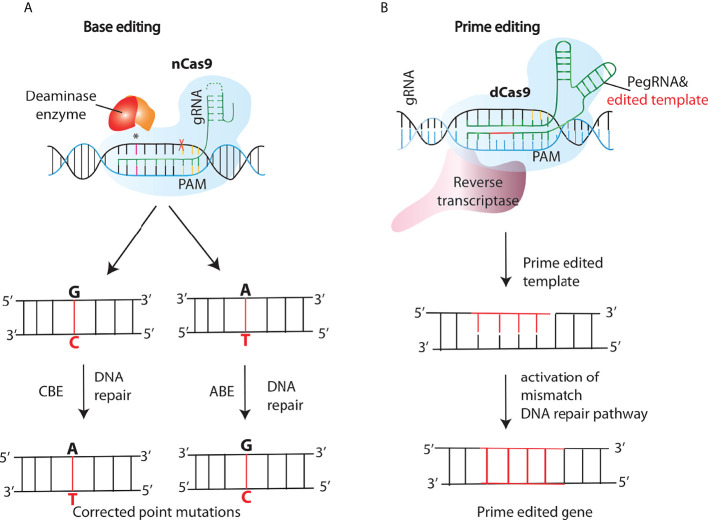
New generations of genome editing tools- base and prime editing. **(A)** Cytosine and adenine base editors (CBEs or ABEs), consist of a nickase Cas9 (nCas9) fused to a deaminase that nicks the opposite strand inducing a DNA repair pathway response, resulting in the conversion of C:G into T:A or A:T into G:C base pairs in the editing site. **(B)** In the prime editing (PE) method, a PE gRNA (pegRNA) complexes to the fused retroviral reverse transcriptase (RT)- nCas9 complex, tethering it to the target site. After the nicking of the target strand by nCas9, the RT synthesizes a new DNA strand using the sequence included in the pegRNA as a template, and thus introducing the desired edit.

A significant number of human blood genetic disorders are due to point mutations or single nucleotide polymorphisms (SNPs) compelling the need to reverse these mutations without generating indels which are an inevitable product of DSBs (64). To this aim, Base editors (BEs) were developed as a new approach of genome editing that change one target DNA base into another in an irreversible and programmable manner. This new class of genome editing tools is capable of correcting many different types of mutations without producing DSBs and has been shown to treat disease-associated point mutations in mammalian cell lines with a very high efficiency and importantly at the same time keeping the indel formation to a minimum (>0.1-1%) (65–67). Structurally, BEs are engineered proteins made by tethering a catalytically dead Cas9 (dCas9) or a nickase Cas9 (nCas9) fused to a cytidine (or adenine) deaminase and guided by a single guide RNA (sgRNA) to the target site (**Figure 4A**). These fusion proteins have a high appeal in therapeutics as well as biotechnology and are considered safer due to the absence of DSBs and a remarkable reduction in off-target effects (68, 69). Furthermore, to overcome the limitation of single-function base editors (C·G→T·A and A·T→G·C), Sakata et al., developed dual function base editors with both C→T and A→G base substitution activities and importantly with similar on and off-target effects as the single site base editors (67). Base editors have already shown their potential in initial attempts to treat β-thalassemia in patient cells (70) and Progeria-a rare disease caused by a C•G-to-T•A mutation in *LMNA* gene in mice (71).

Another significant addition that further enriched the genome editing toolkit was the development of a “search-and-replace” technology named as prime editing (PE). In contrast to base editing, PE system consists of a prime editing extended guide RNA (pegRNA)-guided reverse transcriptase, instead of a deaminase, fused to the Cas9 nickase (**Figure 4B**). Upon nicking of the target strand by nCas9, the Reverse Transcriptase synthesizes a new DNA strand using the sequence included in the pegRNA as a template, and thus introducing the desired edit. Unlike the existing genome editing methods described above, PE can accomplish not only all 12 types of point mutations, but also the small insertions and deletions, or even the combination of substitutions, insertions and deletions without the need of DSBs (72). The approach was further modified by Anzalone et al., known as twinPE, to enable the insertion, deletion or substitution of larger DNA sequences at target loci (73). Moreover, Engineering of the 2-component system PE2 has resulted in more efficient versions of the PEs, for instance, PE3 contains an extra gRNA to create an additional nick at the target site in order to increase editing efficiencies (72). PE has already been tested in a few genetic disease loci like SCD and Tay-Sachs disease (TSD) which are caused by a point mutation and a 4-bp insertion respectively (72). Base and prime editing have also made it easier to perform *in vivo* genome editing based treatment of genetic diseases which was otherwise challenging. Recently, Bock et al, using an engineered PE lacking an RNAse H domain is able to revert disease-causing mutation targeting liver in a mouse model of phenylketonuria (PKU) (74) Moreover, by combining two PE pegRNAs with a Cas9-RT conjugate, Xue and coworkers developed a novel PE-Cas9 based deletion and repair method (PEDAR) that precisely removed a 1.38-kb pathogenic insertion within the tyrosinemia causing *Fah* gene (Fumarylacetoacetate hydrolase) to restore its expression in the liver of a mouse model of tyrosinemia (75)

## Therapeutic Genome editing for the treatment of Wiskott - Aldrich syndrome

### Overview of genome editing to treat PIDs

As most PIDs are caused by loss of function mutations, the most frequently applied GE approach to treat these disorders relies on the activation of the HDR pathway for either correction of the mutations or site-specific addition of a correct copy of the faulty gene. The first approach, using single strand oligo DNAs (ssODN) as homology templates, has been shown to be the most efficient and straightforward between the two, however it requires expensive and time-consuming tailoring of the GE platform to each single patient, given that most PIDs are caused by a wide range of mutations spread across the faulty genes. As such, targeted gene insertion is considered the preferred methodology to tackle PIDs, providing a one size-fits all approach that could be applied to all the patients affected by a specific disease (76) (**Figure 3B**). HDR can be harnessed to insert a gene in its own genomic locus, resulting in its tight, physiological expression regulated by endogenous regulatory elements, into ‘safe harbor’ regions (77), or in highly transcribed loci to achieve strong and/or tissue specific protein expression (78). The delivery of full-length open reading frames flanked by homology arms as homologous templates for HDR to primary cells is more challenging compared to that of small ssODN; while first attempts favoured the use of integrase-deficient lentiviral vectors (IDLV) for that scope, recently the field has transitioned to non-integrating AAVs as more efficient delivery vehicles of homology templates into HSPCs. Many studies in recent years have shown successful targeted integration in HSPCs of corrective genes involved in various PIDs, such as X-linked Severe Combined Immunodeficiency, Chronic Granulomatous Disease, X-linked Hyper IgM Syndrome, Wiskott - Aldrich syndrome and X-linked Agammaglobulinemia using the aforementioned strategy (79, 80). While the first pioneering studies targeting *IL2RG* (81, 82) reported only negligible rates of GE-mediated targeted integration in primary cells, the field has hugely benefited from the implementation of optimized cell culture protocols, delivery methods and GE reagents, resulting in >80% allele correction rates in edited HSPCs reported in more recent attempts. To overcome some of the limitations associated with the use of AAV vectors as carriers of DNA template in primary cells (83) scientists are exploring different non-viral delivery strategies including Virus-Like Particles (VLPs) combined with a DNA donor template (84) or double- and single strand DNA formulations (85), with however limited efficiency obtained in HSPCs so far.

### Genome editing to treat WAS

WAS arises from >400 different genetic mutations scattered throughout the *WAS* gene, with 9 mutational hot spots accounting for one third of all mutations detected so far (24) (**Figure 1A**). As such, the most cost and time effective strategy to treat WAS through GE is to insert a correct copy of the WAS gene, or portions of it, into the genome, thus providing a universal platform amenable to all WAS patients. The first study for the use of GE to tackle WAS has been reported by Laskowski et al. in induced pluripotent stem cells (iPSCs) derived from a WAS patient (86). In this study, ZFNs targeting intron 1 of *WAS* were delivered into the cells in the form of plasmid, together with a corrective WAS sequence spanning exon 2 to exon 12. The authors reported insertion of the corrective cassette at unknown frequency, physiological levels of WASp expression and differentiation of iPSCs into functional T and NK cells. A second study published two years later by Gutierrez-Guerrero et al. used a similar strategy to introduce *via* HDR a GFP reporter cassette into the *WAS* first intron in K562 cells (87). The authors compared the efficiency of ZFN and CRISPR/Cas9-based platforms delivered by plasmid transfection or IDLV transduction, achieving up to 6% of targeted integration when using the viral delivery platform. Both studies provided early and fundamental proof-of-concept of the feasibility of site-specific gene knock-in into the *WAS* locus; however, they suffer from several limitations from the therapeutic point of view, including the use of cell lines, the observed frequency of targeted integration being much below the curative threshold and the use of strategy that would not be amenable to WAS patients bearing mutations in *WAS* exon 1.

Our group has recently overcome these limitations by developing a highly specific and efficient CRISPR/Cas9 platform paired with an AAV6-based homology template delivery system to insert a codon optimized *WAS* cDNA in frame with its endogenous translation start codon in exon 1 in HSPCs derived from healthy donors and WAS patients (80). With optimized protocols, we showed knock-in of a GFP reporter cassette in up to 69% of HSPCs, without compromising cell viability and colony forming potential. To test the ability of our gene editing protocol to restore functional WAS expression, WAS patient-derived HSPCs were electroporated with Cas9/gRNA and transduced with a therapeutic AAV6 donor vectors carrying the WAS cDNA flanked by homology arms. In parallel, cells were also transduced with the LV currently used in WAS GT clinical trials to compare protein expression with the two different strategies. We demonstrated that the delivery of editing reagents to WAS HSPCs led to the integration of the therapeutic cassette in the *WAS* locus and to full correction of all functional defects in B- and T- cells, macrophages and most importantly of platelets both *in vitro* and *in vivo*, which was achievable by virtue of a regulated and physiological expression of WASp in all lineages. WAS cells transduced with the clinical grade LV at one copy per cell showed an improved morphology and functionality, but to a much lesser extent compared to gene edited cells, with limited correction of B-cells, platelets and myeloid cells, possibly due to sub-optimal WASp expression. Primary and secondary transplantation of edited HSPCs into immunodeficient also confirmed the preservation of their repopulating potential and persistence of corrected cells *in vivo*, without any noticeable toxicity. Although further safety and efficacy data coming from ongoing preclinical studies (88, 89) are necessary to endorse the feasibility of this system and its therapeutic value in the context of WAS, our and previous work have paved the way for an alternative, yet highly efficient, safe and precise treatment for this disease.

## Outstanding challenges in the field of HSC gene editing

Despite the above-mentioned advances in the field and the tremendous improvements in different aspects of HSPC manipulation that have been obtained recently, there are still major challenges that need to be addressed to ensure a safe and efficacious translation of GE into the clinics. Here we summarise what we believe are the three major areas of improvements in the case of GE applied to HSPCs.

### Nuclease specificity

Despite their ability to induce targeted modifications, early applications of GE platforms to cell lines and primary cells generated significant off-target effects (OTEs), defined as DNA cleavage events at non-target loci (90, 91). In the case of the most frequently used CRISPR/Cas system, these events usually occur as a result of the Cas9 protein occasionally tolerating around 1-5 mismatched bases between the gRNA and DNA sequence, which is thought to be an adaptation for maintaining immunity against mutated bacteriophages (92). OTEs are a significant problem in translational PID research and because installing unwanted mutations in the therapeutic cellular target, HSPCs, can result in their long-term reproduction throughout the entire hematopoietic system, potentially producing many unwanted physiological changes. Oncogenic mutations are of greatest concern, but mutations at other genomic locations may as well lead to undesired effects. To prevent the risks associated to OTEs, comprehensive detection and analysis of the widespread activity of genome editors are mandatory. Screening can be conducted *in silico*, *in vitro* and recently even *in vivo*. These approaches must be used in a complementary fashion as none provides comprehensive site-specific OTE detection/prediction and they differ in their domains of sensitivity. Predictive algorithms benefit from speed and ease of use but can fail to give truly genome-wide predictions (93, 94). Genome-wide detection methods reduce this bias but are usually conducted in cell lines with little clinical relevance (e.g. IDLV trapping and GUIDE_seq), or *in vitro* on Cas-digested genomic DNA (e.g. DIGENOME-seq and CIRCLE-seq) hampering the applicability of their findings to the relevant cell type or in clinical settings. They also suffer from insufficient sensitivity towards low-frequency off-target events (95) (. One strategy for making the most of these technologies is to combine biased and unbiased methods to generate a predicted list of OTE sites, then edit the cell type of interest and use NGS to confirm OTEs (96). Caution is advised in discarding predicted OTEs as NGS can only detect mutations present in at least 0.1-0.01% of cells. Recent findings have proved the feasibility of off-targets quantification *in vivo* (97), however a robust and reliable pipeline to assess off-target fluctuation in HSPCs during manufacturing and after transplantation in clinical settings has not been established yet. In parallel to the installation of unwanted and unspecific mutations in the genome, GE carries the risk of inducing gross chromosomal translocations resulting from fusion of on- and off-target cutting, as well as of chromothripsis, occurring as a consequence of on-target editing (95, 98). These large aberrations have been detected post-transplantation *in vivo* in large animals and humans (99, 100), and despite they have not resulted in the development of oncogenesis yet, their presence in the infused product has to be carefully evaluated to avoid safety concerns. The landscape of nuclease specificity is further complicated by the fact that once an OTE is detected, predicting its potential impact on cell fitness is all but trivial and requires the development of functional readouts of safety specific to the therapeutic cell type of interest. This is particularly cumbersome for HSPCs, given the paucity of reliable *in vitro* mutagenesis assays and of *in vivo* models of oncogenesis that also allow transplantation of human cells.

Alongside the development of sensitive tools for the detection of OTEs, the field has quickly progressed with the design of safer GE reagents. Cas9 proteins were rationally designed to generate early high-fidelity variants with reduced non-specific DNA affinity (101, 102) and increased energy barriers to DNA cleavage (92). Sophisticated directed evolution screens followed which iteratively improved both on-target efficiency and OTE rates (103–105), most notably the development of HiFi-Cas9 (106). HiFi-Cas9 is an excellent editor owing to a single mutation in the recognition lobe which is expected to increase the energetic barrier to DNA cleavage such that it is generally insurmountable when the gRNA and candidate genomic sequence bear any mismatches. A recent systematic study confirms that HiFi-Cas9 has a superior on-target activity to specificity (i.e. incidence of OTEs) ratio relative to all other high-fidelity Cas9 variants currently available (107). HiFi-Cas9 has robust activity at clinically relevant loci in HSPCs with 15-fold fewer OTEs than WT SpCas9 (106). Concurrently, HiFi-Cas9 delivered as a ribonucleoprotein (RNP) shows excellent activity and specificity in recent *ex vivo* PID gene therapy research, indicating its potential as a mainstay of the field (80) ). Due to its reasonable specificity even with continuous expression in HEK293 cells, it may in future lend itself to *in vivo* delivery *via* viral vectors, nanoparticles or linkage to tissue-specific delivery peptides. It is worth considering however that different SpCas9 variants tend to have different efficiencies in different editing contexts, for example at repetitive target sequences (101). Base editors consist of a toolkit in which each is optimised for activity on a highly restricted set of edit site types. Each editor therefore has a far higher efficiency and specificity for its specific editing context than a general editor would. This strategy has recently been entering high-fidelity Cas9 research, such as with the development of LZ3-Cas9, optimised specifically for generating single base insertions and exhibiting superb activity and specificity relative to generalist high-fidelity Cas9 proteins (107). PID research may in future benefit from the use of an HSPC-validated toolkit of high-fidelity Cas9 proteins specialised for different editing contexts, allowing the best possible editing activity and specificity in each disease context.

### Efficiency of LT-HSCs correction

An effective HSPC-based therapy must include editing of both long-term hematopoietic stem cells (LT-HSCs) and progenitor cells. LT-HSCs are capable of both self-renewal and differentiation into all hematopoietic cell types but are generally quiescent; in these therapies, they replenish corrected blood cells in the long term. Progenitors have a reduced capacity for self-renewal and are committed towards specific hematopoietic lineages but proliferate more rapidly and so provide hematopoietic reconstitution in the early stages following transplantation (108). GE approaches have shown preferential genetic correction of the prevailing population of more committed progenitors, at the expense of rarer LT-HSCs, when manipulating HSPCs *ex-vivo*. The lower frequency of gene correction by HDR in LT-HSCs being elicited by a combination of different components, including, the inefficient delivery of the donor template by viral transduction, the quiescent nature of LT-HSCs which reduces the activity of the HDR pathway, and the intrinsic cytotoxicity of the procedure. To enhance the total DNA template load in the nucleus of cells, scientists have turned to more efficient donor delivery methods, such as AAV6 with higher tropism form HSPCs, or to the use of cyclosporin H which increases the transduction of stem cells when lentiviral vectors are used as HDR donor templates (109). Other strategies have been adopted to increase the HDR efficiency by transiently manipulating the DNA repair pathways, favouring HDR over NHEJ for example, however with limited improvements in HSPCs (110, 111). The most successful approach so far has probably arisen from the attempt to promote LT-HSC cell cycle progression to increase the engagement of HDR components and hence the knock-in exogenous sequences, which has been accomplished by the use of cell cycle modulators (112, 113). Overall, the implementation of these methods to circumvent GE limitations have led to outstanding levels of gene engineering in HSPCs and LT-HSCs *in vitro*, with >80% of correction rates achieved with GE in HSPCs, predicted to be curative for many inherited blood disorders. However, while these strategies have confirmed efficacious, they in turn entail a new set of impediments to overall success of the therapeutic intervention (see below).

### Engraftment of corrected LT-HSCs

Despite the excellent levels of correction of HSPCs and LT-HSCs currently attainable *in vitro* and the maintenance of their transplantability into immunodeficient NSG mice or non-human primate models, recent studies have shown that animals transplanted with *ex-vivo* manipulated HSPCs display an overall lower human engraftment rate than those infused with unmanipulated control cells, and this is particularly true when performing HDR-mediated gene correction. More importantly, scientists have also witnessed a decrease in the frequency of engrafted corrected cells over the course of the 16-30 weeks long transplantation studies in mice and clinical trial follow-ups in patients. This is an important and pressing issue for the GE field, as inadequate engraftment and persistence of corrected cells hampers the broader application of these technologies to the treatment of blood disorders that require high chimerism post transplantation and that do not display strong selective advantage of corrected cells. There is good evidence that reduction of the frequency of corrected cells *in vivo* is negligible early after transplant, but becomes prominent at later stages, suggesting that the cause lies within the population of LT-HSCs as an effect of either their limited correction, limited presence in the population infused or their limited engraftment and self-renewing ability post manipulation.

The decreased rate of gene edited cells *in vivo* observed in animal experiments and the delayed engraftment of myeloid cells and platelets observed in gene therapy-treated patients (114) has been partially counteracted by increasing the doses of HSPCs to be transplanted. Not only has this approach brought only limited improvements in the context of HDR-gene edited cells, but it is also not always feasible in patients that respond poorly to the mobilization regimen (poor mobilizers) and it comes with increasingly high product manufacturing costs. Several studies and possible solutions have emerged in the past couple of years by focussing on devising ways to preserve LT-HSCs during the *ex-vivo* manufacturing process, in order to ensure their abundance in the infused cellular product and the maintenance of their long-term repopulating characteristics *in vivo*. Indeed, it is well known that prolonged culture and stimulation of HSPCs, while being needed for efficient gene transfer, may adversely impact their engraftment and long-term repopulation capacity. Recent findings showed that addition to the culture medium of stemness preserving compounds, such as Stem Regenin-1, UM171, and 16,16-dimethyl prostaglandin E2 (dmPGE2), helps to maintain the long-term multilineage repopulation capacity of human corrected HSPCs transplanted in immunodeficient mouse models, partially overcoming the drawbacks of prolonged culture (96) and that fine-tuning of cytokine composition can lead to a beneficial balance between preservation of stemness and cell expansion (115, 116). Another aspect that needs special attention is the tolerability of HSPCs to genetic modifications; indeed LT-HSCs, which require protection from mutational load over a lifetime, are more sensitive to DNA manipulation than differentiated cells, hence GE may trigger cellular responses that reduce their fitness and stemness. It has been shown that exposure to GE reagents, particularly in the case of HDR-mediated gene addition, activates an innate interferon immune response and a p53-dependent DNA damage response that leads to cell cycle arrest, senescence, or apoptosis which in turn lead to HSC differentiation and exhaustion of their long-term repopulating potential (117). Dampening of the DNA damage repair pathway by use of p53 inhibitors has shown sensible improvement in both increasing the frequency of corrected LT-HSCs and their engraftment in mice, although it may carry the risk of selecting populations with chromosomal abnormalities. Despite their minimal genomes, AAV6 vectors can trigger innate immune responses in HSPCs *via* innate immune receptors such as Toll-like receptor 9 (TLR9) which senses microbial dsDNA containing unmethylated CpG motifs, and TLR2 (118, 119). TLR9 activation induces a type I interferon response and inflammatory cytokines, impacting on HSPC survival. One approach to reduce the residual immunogenicity of AAV6 vectors is to ‘humanise’ them by removing pathogen-associated molecular patterns (PAMPs) like CpGs. A risk factor calculation tool has recently been developed to assess AAVs for TLR9 activation and assist researchers in redesigning AAV constructs to reduce immunogenicity (120). Another approach is to embed TLR9-inhibitory oligonucleotides into AAV genomes, and this has been demonstrated to reduce innate immune responses in mouse and pig models (121). TLR9-inhibitory oligonucleotides can also be co-administered with nonviral vectors such as ssODNs.

## Gene editing versus gene therapy in the context of WAS

Being built over the very same procedure and preclinical protocols used for *ex-vivo* LV GT of WAS, GE entails the same advantages of GT over HSCT and could soon represent a valuable alternative to existing therapeutic strategies. While HSCT represents the gold standard for those patients who have access to matched donors, a question on which gene correction strategy to use as a second line of treatment could be raised when HSCT is not a viable option. Both GT and GE have their own set of advantages and drawbacks which must be carefully weighted. In the case of WAS, LV GT has the undisputed advantage of being a more advanced approach, as it has already undergone a thorough safety and efficacy scrutiny in preclinical and phase I/II clinical studies and additionally it can benefit from a broader experience of GT approaches for other PIDs and blood disorders. However, as previously discussed, clinical studies on LV GT have highlighted an incomplete alleviation of the WAS phenotype with, most notably, limited improvement in platelet counts in a number of patients, and generally lower levels of correction within the B-lymphocyte and myeloid compartment, thereby risking persistence of inflammatory complications and autoimmunity (41–43, 45). As clinical improvement is dependent on high levels of viral marking, a wild-type WAS expression is essential to completely and reliably correct the disease phenotype in all affected lineages. Evidence collected from WAS patients treated with GT (43) and by us (80) suggest that the limited improvement in myeloid correction could be a consequence of the sub-physiological levels of WASp expression mediated by the therapeutic LV. With LV, achieving sufficient transgene expression depends primarily on the choice of the internal promoter; a weak promoter may result in insufficient protein expression and limited functional rescue, whereas a strong promoter may lead to overexpression and increased risk of toxicity or insertional mutagenesis. Current clinical trials for WAS utilize a LV with an internal promoter consisting of a 1.6 kb fragment of the endogenous WAS promoter to drive human WASp expression, which may be insufficient to recapitulate full expression in certain hematopoietic lineages. As a means to overcome these limitations, different research groups have tried to: 1) potentiate the current WAS promoter used in LV gene therapy by identifying and introducing WAS endogenous enhancer sub-regions to the existing vector design, with however little or no improvement in the level of protein expression (122); 2) substitute WAS endogenous promoter with a strong synthetic viral promoter; while this strategy successfully restores WASp expression, it has important safety issues as it aggravates the risk of genotoxicity due to insertional mutagenesis (123). In this regard, a GE strategy that aims to integrate a correct WAS gene in its own locus represents an important advantage, as it ensures physiological transgene expression from all WAS regulatory regions (80), which could be located far away from the promoter, too big to fit into a lentiviral cassette (122), or yet undiscovered. The strategy would also exclude uncontrolled protein overexpression from strong promoters included in viral vectors or sub-physiological protein expression caused by weak synthetic promoters or by DNA methylation-mediated promoter inactivation (124). Data stemming from our proof-of-concept study of GE applied to WAS hinted to full reconstitution of WASp expression contributing to more reliable correction of thrombocytopenia; if confirmed by further *in vivo* studies, this evidence could enable a broader application of GE also to patients with attenuated WAS who predominantly manifest thrombocytopenia and bleeding risk and who are excluded from GT applications because of limited benefits.

Controlling transgene integration entails additional advantages. First of all, it guarantees the maintenance of a normal gene copy number in the therapeutic cell of interest. In GT clinical trials, WAS patients were infused with a HSPC bulk carrying an average of 2 vector copies per cell, to ensure sufficient transgene expression from the LV internal promoter and therapeutic benefit, which however was not achieved in all hematopoietic lineages (41). While the introduction of transduction enhancers in HSPC manufacturing protocols (125, 126) could further boost cell transduction and consequently therapeutic protein expression, the integration into the genome of many LV copies could potentially increase the risk of insertional mutagenesis. On the same line, targeted insertion of the transgene *via* GE avoids the risks associated with the semi-random integration pattern of LV and potential genotoxicity arising from transcriptional deregulation and aberrant splicing (127, 128). This is particularly important in the case of WAS, where patients are predisposed to malignancies and clonal expansion. Although such risks have not been observed in patients treated with LV GT so far, long-term clinical follow ups to monitor delayed adverse events are desirable in order to fully confirm the safety of these vector designs. On the other hand, safety is of great concern also for the GE counterpart, where installing unwanted mutation at off-target sites could potentially lead to the development of cancerous lesions. Despite the development of safer nuclease versions and despite preclinical data for the WAS CRISPR/Cas9 GE platform showing no major genotoxicity issues (80, 89), existing technologies to detect off-targets and to assess functional consequences in the target cells are not exhaustive enough and clinical safety data retrieved from patients treated with GE are still scarce. Hence further studies are required to understand the genotoxicity of GE-based therapeutic strategies and compare it with existing technologies before decreeing its potential superiority.

The most undisputable aspect to take in consideration in the GT versus GE diatribe is the therapeutic efficacy of the strategy. Provided that a stable and regulated expression of WASp is achieved, one should also consider the fraction of HSPCs and of more primitive LT-HSCs that can be genetically corrected with either platform to predict a life-long therapeutic benefit. Clinical data derived from WAS patients treated with HSCT (29, 32) suggest that while a lower frequency (<10%) of corrected HSPC engraftment is required to reverse the WAS T cells phenotype, due to their selective advantage once WASp is restored, a much higher percentage of myeloid chimerism (>50%) is required to neutralize the platelets defects. With GT, follow-up data from clinical trials showed that this threshold chimerism is achievable in principle, although not in all the patients. In our proof-of -concept study of GE applied to WAS, high rates of gene correction were achieved *in vitro*, which however declined to 16% in secondary transplants in NSG mice. While one could rightly argue that NSG mice are not a perfect model to assess therapeutic efficacy of the platform and that ultimately this can be assessed only in humans, the limited engraftment of gene edited HSPCs is a phenomenon that has been widely observed in the field, especially when performing HDR-mediated correction, as discussed in the previous chapter (129). As such, reaching sensible rates of corrected cells post-transplantation still represents a significant challenge for GE that limits its application to only those diseases that require either low therapeutic levels of corrected protein or that show very strong positive selection of corrected cells, as in the case of SCID. Even for those eligible diseases, one should also consider that the small number of treated LT-HSCs that efficiently engraft *in vivo* raises the chance of an oligoclonal composition of the corrected graft, which may predispose to its early loss or clonal exhaustion; this in turn represents a risk factor for the emergence and expansion of abnormal clones bearing gain-of-function mutations and hence for the occurrence of genotoxic adverse events in treated patients (114).

Overall, the limited but encouraging evidence available for WAS suggest that both GT and GE are promising and potentially viable therapeutic options for this disease; continued clinical monitoring as well as vector design improvements for GT and more detailed late-preclinical/early clinical data on safety and efficacy for GE are desired to confirm the potential of one or both of these technologies being the second-best line of treatment for patients suffering from classical or mild WAS.

## Concluding remarks

The field of genome engineering has equipped the scientific community with the ability to artificially modify genetic information thereby unleashing the potential of conventional medicine to new therapeutic approaches for a myriad of conditions including metabolic, hematopoietic as well as immunodeficiencies, including WAS. As the search for new and safer tools is progressing at an incredible pace, it is highly likely that more additions will be made to the toolkit; nonetheless efforts are needed to address the safety and specificity of these tools further and thoroughly. To treat or cure WAS by means of genetic correction it is of paramount importance to match the underlying genetic defect with the plethora of genetic engineering strategies and platforms available. Before favouring a particular tool, few considerations should be contemplated such as the type of editing needed, the delivery method required, and the extent of gene repair or correction essential to achieve clinical benefit.

## Author contributions

AN, ZS and AC wrote the original draft and AN prepared the figures. AC supervised and edited the article. All authors contributed to the article and approved the submitted version.

## Funding

This manuscript was supported with grants from the Great Ormond Street Hospital Charity and LifeArc Translational Research Accelerator Grant (VS0420), the UKRI Medical Research Council (MR/W001314/1) and the National Institute for Health Research Biomedical Research Centre at Great Ormond Street Hospital for Children NHS Foundation Trust and University College London.

## Conflict of interest

The authors declare that the research was conducted in the absence of any commercial or financial relationships that could be construed as a potential conflict of interest.

## Publisher’s note

All claims expressed in this article are solely those of the authors and do not necessarily represent those of their affiliated organizations, or those of the publisher, the editors and the reviewers. Any product that may be evaluated in this article, or claim that may be made by its manufacturer, is not guaranteed or endorsed by the publisher.
